# Evaluation of the performance of a chlorhexidine gel containing CVC dressing in a clinical environment

**DOI:** 10.1186/2047-2994-4-S1-I1

**Published:** 2015-06-16

**Authors:** T Karpanen, A Casey, T Whitehouse, P Nightingale, I Das, TS Elliott

**Affiliations:** 1 Clinical Microbiology, University Hospitals Birmingham NHS Foundation Trust, UK; 2 Department of Anaesthetics and Intensive Care, University Hospitals Birmingham NHS Foundation Trust, UK; 3 Wolfson Computer Laboratory, University Hospitals Birmingham NHS Foundation Trust, UK; 4 Corporate Division, University Hospitals Birmingham NHS Foundation Trust, UK

## Introduction

Although infection risk associated with central venous catheters (CVC) has reduced in recent years, the use of CVC are still associated with relatively large number of infections, leading to increased patient morbidity and healthcare costs. A major source of microbial colonisation and infection of short term CVC is the patients’ endogenous skin microorganisms located at the catheter insertion site.

## Objectives

To evaluate the introduction and performance of a chlorhexidine (CHG) gel CVC dressing in a critical care environment.

## Methods

Following Ethical committee and Trust approvals and staff training, a transparent film-dressing incorporating an aqueous CHG gel was introduced to critical care patients over a 9 month period. Skin reactions to the dressings and performance characteristics of the dressings were monitored. Any adverse events were determined as per standard clinical practice. Healthcare workers’ perceptions of the performance of the dressing were evaluated at the end of the study period.

## Results

There were no reports of severe contact dermatitis associated with the CHG or standard dressings. Close assessment of skin condition at the CVC site was evaluated in 273 patients who had given their consent. Following dressing removal, mild redness under the adhesive was reported in one standard dressing group patient (0.7%, n=137) and in seven CHG dressing group patients (5.1%, n=136). Only one patient presented with mild redness under the CHG gel part of the dressing (0.7%, n=136). All the above symptoms resolved within 24 h following dressing removal.

A questionnaire was distributed to critical care nursing staff and clinicians in theatres, who had experience handling and observing both the standard CVC (Tegaderm IV dressing) and CHG gel containing CVC dressing (CHG Tegaderm). In total, 71 nurses and 10 clinicians responded to the survey. Staff was satisfied with the performance of the CHG dressing, with 97.5% of the respondents rating the overall performance of the CHG gel dressing as: the same as (11.1%), better (35.8%) or much better (50.6%) than the standard CVC dressing.

## Conclusion

The CHG gel CVC dressing was well tolerated by patients and performed effectively in the critical care environment.

## Disclosure of interest

T. Karpanen Grant/Research support from: received funding for attending the ICPIC (2015) conference, A. Casey: None declared, T. Whitehouse: None declared, P. Nightingale: None declared, I. Das: None declared, T. Elliott Speaker's bureau of: received honoraria for presentations at symposia, Consultant for: has received honoraria for attendance at advisory board meetings.

